# Beyond the Literal Meaning of Words in Children with Klinefelter Syndrome: Two Case Studies

**DOI:** 10.3390/brainsci8090171

**Published:** 2018-09-07

**Authors:** Sergio Melogno, Maria Antonietta Pinto, Margherita Orsolini, Luigi Tarani

**Affiliations:** 1 Department of Psychology of Development and Socialization Processes, “Sapienza” University of Rome, 00185 Rome, Italy; mariantonietta.pinto@uniroma1.it (M.A.P.); margherita.orsolini@uniroma1.it (M.O.); 2 Pediatric Department, “Sapienza” University of Rome, 00185 Rome, Italy; luigi.tarani@uniroma1.it

**Keywords:** Klinefelter Syndrome, pragmatic skills, figurative language, assessment, developmental trajectories

## Abstract

Literature on children with Klinefelter Syndrome (KS) points to general linguistic difficulties in both comprehension and production among other cognitive functions, and in the majority of cases, these coexist with an intellectual level within the norms. In these conditions, children having language delay generally engage in language therapy and are systematically monitored across ages. In this article, we present the profiles of two children with KS (47, XXY), aged 9.1 (Child S) and 13 (Child D), whose language development was assessed as adequate at age 3, and for this reason, did not receive any language treatment. At the present stage, their IQ, as measured by Wechsler Scales (Child S: 92; Child D: 101), is within the norm, but they both present marked weaknesses in pragmatic skills such as figurative language comprehension. The analysis of these two cases points to the need to go beyond global indexes of verbal abilities, as the same global index may mask a wide diversification of individual profiles. In addition, this study underlines the importance of monitoring the developmental trajectories of children like Child D and Child S, because weaknesses in pragmatic skills that are relevant for both academic achievement and social adaptation could emerge at later stages.

## 1. Introduction

Klinefelter syndrome was described for the first time by Klinefelter and colleagues [1] in the early 1940’s as a genetic anomaly due to the presence of a supplementary X chromosome in males, who, for this reason, have a 47, XXY kariotype instead of the usual 46, XY. While the typical kariotype is 47, XXY, which represents 80% of cases, there may be also other kariotypes such as 48, XXXY, 48, XXYY, 49, XXXXY, 46, XX, or 47, XXY (the remaining 20%). This syndrome, which is the most frequent gonosomic anomaly, has an incidence of 1:600 male born, with a quite variable clinical pattern, largely under-diagnosed. In fact, the characteristic hypogonadic eunuchoid phenotype may be observed less frequently than an almost normal phenotype, characterized by the only constant clinical signs of microrchidia (small testicles) (<10mL in adulthood) and azoospermia (absence of spermatozoa in the seminal fluid) that can be detected in adulthood, especially in cases of infertility. Pediatric clinical signs are even rarer and non-specific, but suspicion can be advanced in the case of cryptorchidism (a condition in which one or both testicles fail to move from the abdomen, where they develop before birth, down into the scrotum), hypogenitalism (subnormal development of genital organs), hypospadias (an abnormality of the penis in which the urethra opens on the undersurface), language delay, and particularly the length of the lower limbs, compared to upper limbs, within a eunuchoid habitus. Puberty is to be evaluated by the low volume of testes (<3mL at 11 years), pubertal delay, modest development of secondary sexual characters, and the appearance of gynecomastia that may confer, in particular overweight patients, a gynoid appearance for the deposition of fat to the root of the arts. Clinical diagnosis is then confirmed by the examination of the karyotype and the hormonal dosage (hypergonadotropic hypogonadism). Medical therapy consists in hormone replacement with testosterone when androgens values are insufficient. 

The majority of individuals with KS have intelligence levels within the norms, with some cases of intellectual functioning at the border, and of intellectual disabilities in 3–4% of cases—as it is in the general population [2]. Great variability is attested and the intellectual profile may change across a lifespan [3]. Additionally, the neuropsychological profile can be extremely heterogeneous [4], where strengths are to be found in non-verbal abilities and weaknesses in motor skills, attention, executive function (inhibition, working memory, verbal fluency, etc.) [4,5,6], language, and basic literacy competencies [3,7]. Language development is reported as delayed [8,9,10,11], and tend to be under the general intellectual level.

Bishop and Sherif [11] found interesting similarities between children with specific language impairment and the language profile of children with KS. However, expressive deficits are more pronounced than the receptive ones [12]. In a study on a sample of 50 children and adolescents with KS (47, XXY) (age range: 4.1–17.8), based on a wide spectrum assessment, Ross and colleagues [3] analyzed the evolution of the linguistic and the entire neuropsychological profiles. At all ages, language and speech impairments remained evident in some form. Of particular interest, in relation to the focus of our study, is the domain studied by the authors. By using the Wiig and Secord’s [13] Test of Language Competence-Expanded Edition (TLC-E), Ross and colleagues explored four areas: Ambiguous Sentences, Listening Comprehension, Oral Expression, and Figurative Language. In particular, in Ambiguous Sentences, the participant (P) is presented with an ambiguous sentence where the same word can be interpreted in at least two different ways. In Figurative Language, P is presented with a sentence in a given context where some lexical unit (e.g. adjective) is used metaphorically. P is then requested to explain the meaning in his/her own words. Afterwards, four possible solutions are presented visually and P must choose what he/she thinks is appropriate. The results showed that the total sample had an average score of −1 standard deviation at the composite TLC-E score, although there were variations across ages. The youngest children (under the age of 10) had average performances in Figurative Language, but not in Ambiguous Sentences. Whereas older children (above the age of 10) performed lower in both tasks and the differences between the two age groups were statistically significant.

In our study, we also explored pragmatic skills [14], viewed as those competencies that enable individuals to appropriately interpret meanings embedded in discourse, and as a consequence, to interact adequately in social and cultural contexts. In other words, this ability requires the subjects to infer appropriate information from implicit discourse beyond the literal meaning of words. An example of a social situation requiring a series of inferences is the following dialogue between three characters: “You know I don’t like carrots.” Said Mark (Character 1), standing up from his chair. Character 2 (undefined) says: “Don’t worry, I can bring you something else”. Louise (Character 3), while giving Mark the keys of the car, says (in a low and irritated tone): “If you had to behave this way, why didn’t you remain at home?”. To be able to understand what happened, a child must infer: (a) That the scene takes place in a restaurant, where Character 1 has received a dish he doesn’t like and reacts impolitely; (b) that Character 2 is a waiter; (c) that Character 3 is acquainted with Character 1 and considers his behavior impolite; and (d) that, presumably, Character 1 will come back home by car. This example is taken from a subtest of the APL Medea [15] that has been used in the present study. The examiner reads this social situation with a different intonation according to the different characters. To be able to understand the implicit information and integrate it in a consistent picture requires going beyond basic language competencies (relying on grammar rules and vocabulary) and to decode subtle linguistic cues such as prosodic variations, and also the world knowledge and perspective-taking or Theory of mind [14,16,17].

Another typical capability which requires a complex inferential process in order to find unexpressed meanings under the surface of expressed meanings is figurative language capability, metaphor in particular. For instance, the metaphor “That child is a train without a locomotive”, phrased in the classical ‘X is Y’ form, requires to rephrase “child” (X) as an uncontrolled human being, and “train without a locomotive” (Y), as an uncontrolled physical object, and to find a commonality between the two relevant enough to justify this association. Actually, this common ground is a new meaning resulting from a comparison. Various factors can be held responsible for the development of such complex inferences. Among these, encyclopedic knowledge about each term of the metaphor [18], Theory of Mind [19], executive function, such as inhibition of literal meanings, and working memory to keep the characteristics of both ‘X’ and ‘Y’ in mind [20,21]. Metaphor comprehension, and in general, figurative language comprehension, have been viewed as advanced tests of Theory of mind [19]. Actually, going beyond literal meaning requires inferential processes, consisting of a series of mental operations that allow us to draw plausible conclusions based on the available information. In addition, to infer what is not explicitly said requires a mentalization capability, i.e., to infer the interlocutor’s communicative intention. Not surprisingly, experimental research found a significant association between the capability to solve first and second order false belief tasks and the comprehension of various forms of figurative language, including metaphor and irony [19]. Recently, Theory of Mind capabilities have been explored in individuals with KS and were identified as an area of weakness [22,23]. The plurality of these factors and their relevance easily explains why a possible weakness in one or more of them can negatively affect metaphor comprehension.

This case study describes the profiles of pragmatic skills and the Theory of Mind of two children (Child S and Child D), aged 9.1 and 13, respectively, prenatally diagnosed with KS (47, XXY). As their language development was found to be adequate at the age of 3, they did not receive any language therapy. The aim of our study was to consider the status of pragmatic skills in a critical perspective. The discussion will analyze some implications for future research into the developmental trajectories of this type of child, which is relevant from both a theoretical and application point of view.

## 2. The Study

The present study was carried out at the Department of Pediatrics and Child Neuropsychiatry—“Sapienza” University of Rome All subjects and their parents gave informed consent and assent. The clinical evaluation was performed at the same department. The information provided at par. 2.1. reflect what was available in the children’s clinical documentation, which fundamentally consisted in the outcomes of Wechsler scales at different times.

### 2.1. Participants

#### 2.1.1. Child S

Child S was prenatally diagnosed as a child with KS. He is a native speaker of Italian, monolingual, from an average socio-economic family background. From birth to age 3, Child S has been monitored in his motor, linguistic, and cognitive development. At age 3, he was assessed as a typically developing child with respect to phonological, lexical-semantic, and morpho-syntactic abilities. At age 6, assessed with the Wechsler Preschool and Primary Scale of Intelligence (WPPSI) [24], he showed a total IQ of 103, a verbal IQ of 102 and a performance IQ of 104. Based on this assessment, Child S was considered to be a normally developing child, with a homogeneous profile. At age 8, the Wechsler Intelligence Scale for Children (WISC III) [25] confirmed that his overall cognitive level was within norms (Total IQ: 92), but with a discrepancy between verbal and non-verbal IQ (86 vs. 100). Receptive vocabulary, as measured by the Peabody test [26], was 91 and morpho-syntactic comprehension, as measured by a grammatical test [27], was average. At age 9, productive language was reported by the examiners as normally fluent.

#### 2.1.2. Child D

Child D was also prenatally diagnosed with KS. Like Child S, he is a native speaker of Italian, monolingual, and his socio-economic family background is also average. He was assessed in his motor, cognitive, and linguistic development as a normally developing child, and for this reason, not included in a language therapy program. Further monitoring, from preschool to the beginning of primary school, did not report on any type of linguistic nor cognitive issue, but learning difficulties started to emerge during the years of primary school. Specifically, text comprehension represented a vulnerable area [28] in spite of perfectly adequate decoding skills. The domain of numbers and calculation was also adequate. At the age of 11, at WISC IV [29], he obtained an IQ of 87, with the following indexes: Verbal Comprehension Index (VCI): 82; Perceptual Reasoning Index (PRI): 104; Working Memory Index (WMI): 91; and Processing Speed Index (PSI): 82. Two years later, when he was 13 years old, his IQ raised up to 101, and his VCI to 110, whereas PRI, WMI, and PSI remained fundamentally the same (108, 91, 85, respectively).

### 2.2. Instruments and Measures

The following tests have been used for assessment in the present investigation: (1) A developmental Neuropsychological Assessment (NEPSY II) [30]: (a) Theory of mind (part A and B); and (b) Affect recognition; (2) APL Medea (Italian acronym for ‘Abilità Pragmatiche nel Linguaggio’, developed in the research center called “Eugenio Medea”) [15]; and (3) Metaphor Comprehension Test [31].

The Theory of Mind (ToM) subtest is composed of two parts, verbal (A) and contextual (B). In the A part, the child is presented with images and small texts describing social situations that require interpreting mental states, emotions, and the others’ point of view. The child is asked to answer questions about these situations. In the B part, the child is presented with a figure showing a little girl in various social situations. The child must choose among four alternatives the face of the little girl that better matches the emotional situation depicted in each item. Each item is scored one for the correct answer and zero for the incorrect answer.

The Affect Recognition (AR) subtest assesses the capability to recognize expressions of emotional states (happiness, sadness, anger, fear, disgust, and neutral state) in children’s faces, which are represented in photographs. This test is composed of four tasks. In the first one, the child must say whether two faces appearing in two different photographs represent the same emotion or a different one. In the second task, the child must choose two faces expressing the same emotion among three or four photographs. In the third task, the child is presented with five faces, one at the top and four at the bottom. The child must choose, among the four faces at the bottom, which one matches the same emotion represented in the face at the top. In the fourth task, the child is shown a certain face for a few seconds and then he/she must choose two photographs representing the same emotion depicted in the first face. Each item is scored one for the correct answer and zero for the incorrect answer.

The APL Medea [15] measures pragmatic language skills in children aged five to 14. It is composed of five subtests: Metaphor (M), Implicit Meaning Comprehension (IMC), Conversational Strips (CS), Situations (S), and Colors Game (CG).

The Metaphor subtest assesses the capability to understand figurative language and is composed of two parts—each of four items. In the first half, Verbal Metaphors (VM), the examiner asks the child to explain the meaning of non literal expressions, such as “John is a snail”, whereas in the second half, Iconic Metaphors (IM), the child is asked to choose an image that matches the meaning of expressions read by the examiner among four alternatives. Ex.: “He always has his head in the clouds”. Scoring VM: 0 = completely incorrect answer; 1 = partially correct answer; 2 = correct answer); and IM: 0 = incorrect answer; and 2 = correct answer.

The Implicit Meaning Comprehension subtest is composed of three stories presented as a dialogue, each of which includes 14 items. The examiner reads the stories with a different intonation according to the different characters. After reading, the child is asked to answer some questions, the contents of which must be inferred from the information given in the story (see the example in the Introduction). Scoring 0 = completely incorrect answer; 0.5 = partially correct answer; and 1 = correct answer.

The Conversational Strips subtest is composed of four strips where some empty balloons must be filled in such a way as to match the appropriate parts of a dialogue. Scoring 0 = completely incorrect solution; 1 = partially correct solution; and 2 = correct answer.

The Situations subtest is composed of five items that assess the understanding of meanings in relation to a particular communicative context. The examiner reads each expression with a different intonation according to the type of communicative context and asks the child to find the appropriate meaning. Ex.: The examiner proposes the following case: “If I were you, I would be angry”, said Charles, “Forget about it, said John. Was John angry?”

Scoring four out of five items can be assessed as either incorrect (0) or correct (1). For the remaining items, 0 = incorrect answer; 1 = incomplete, not conforming to social rules; 2 = incomplete but conforming to social rules; and 3 = complete and correct.

The Colors Game subtest is based on a game played by the examiner and the child. The child’s task is to explain the rules of the game to a hypothetical mate who doesn’t know it. Specifically, the explanation must: (a) Describe the materials (a table with three boxes, yellow, green, and red); (b) describe other elements—pawns, one for each player at the starting point, dice with two red faces, one yellow and one green face, one with a smiling and one with a black face; (c) the functions of the smiling face (to move one box) and of the black face (to stay still for one round); (d) moving rules (every player casts the dice once and then passes the round to the other player; players can go ahead with the pawn only if the color of the dice is the same as the one of the next box); and (e) aim, the winner is the one who arrives first at the red box.

The nature of the explanations the child gives can be assessed with a four step-scale: 0 = absent information; 1 = information either incorrect or not intelligible even to those who know the game; 2 = understandable information even to those who do not know the game, but incomplete and imprecise; and 3 = complete and precise information.

The total APL has high reliability, as measured by Cronbach’s alpha (0.922), and also a high interrater agreement of 0.992.

The Metaphor Comprehension Test [31] assesses metaphor comprehension by presenting 12 metaphorical items in the ‘X is Y’ form. From a linguistic point of view, such items are novel metaphors. As this type of meaning is not lexicalized it cannot be retrieved in memory; rather, it must be constructed by establishing a relationship between the two terms of the metaphor.

Ex.: “That child is a train without a locomotive”. The examiner reads each item and then asks the child to explain the meaning of the metaphor.

Scoring 0 = a ‘pre-metaphorical’ answer. These include: “I don’t know” answers; refusals (Ex.: “No, it’s nonsense”); literal interpretation; metonymical interpretation (“That child jumped in the train without locomotive”. 1 = identification of just one semantic feature common to both X and Y, but on material grounds (Ex.: “They both run”); 2 = identification of one semantic feature common to both X and Y on psychological grounds, clearly distinct from concrete features (“That child never stops doing things”); and 3 = identification of one or more semantic features common to both X and Y, well focused with respect to the nature of the metaphor in question (“That child is out of control, just as a train without a locomotive.”).

The MCT has high reliability, as measured by Cronbach’s alpha (0.70) and high interrater agreement, as measured by Cohen’s K (0.75 for the 9-year olds and 0.81 for the 13-year olds).

## 3. Results

Table 1 reports raw scores and their transformation into T scores in the Metaphor Comprehension Test or into scalar scores in the NEPSY II subtests.

Both Child S and Child D performed poorly on the Metaphor Comprehension Test. Child S was positioned in the low range (T: 0–30) and Child D in the medium-low range (T: 31–40). In particular, Child S provided 11 answers at zero level and only one at level one, while Child D provided six answers at zero level, and the remaining ones were distributed between level one (2) and level two (4). 

Scalar scores on the two NEPSY subtests showed performances just below the average for Child S (range: 7–6), and much under the expected level (range: 5–4) for Child D in Theory of Mind, and average in Affect Recognition (range: 10–8). 

Table 2 reports raw scores and their transformation into z scores in the APL Medea subtests and the total test.

As can be seen in Table 2, Child S had moderate difficulties in Implicit Comprehension Meaning (−1.17) and clearly low performances on Situations (−2.47), where he obtained the worst score, and Color Games (−2.04). On the contrary, Child D’s profile showed consistent difficulties, close to low, in some areas of the subtest Metaphor (Verbal Metaphors: −2.58; Iconic Metaphors: −1.80) and in the total score (−2.80). The Implicit Comprehension Meaning subtest also showed a weakness (−1.75). Moderate difficulties also appeared in Situations and Colors Game, but definitely less serious than for Child S. Therefore, for each child, the total APL reflects qualitatively different performances.

## 4. Discussion

This study reported on the assessment of pragmatic skills and Theory of Mind in two children, Child S (9.1 years) and Child D (13 years), prenatally diagnosed as children with KS (47, XXY). As their language development was not delayed, they did not receive any language therapy, but were monitored across ages only for intellectual level with composite tasks, yielding composite indexes. Both children always presented average IQs at Wechsler scales, including the Verbal IQ for WISC III and Verbal Comprehension Index for WISC IV, although these two values fluctuated across time.

Our assessment showed an unexpected finding with weaknesses in pragmatic skills in both children.

Specifically, Child S’s low performance on Metaphor Comprehension Test is due to a nearly total predominance of the lowest qualitative level (zero score). Child S provided pre-metaphorical answers of the magic and the metonymical type. For the item “My sister is a butterfly”, he said: “My sister has been transformed into a butterfly, she became a butterfly”. This type of answer simply identifies one term of the metaphor with the other and alludes to the comparison between the two terms, which is necessary to generate an appropriate interpretation. This type of answer appears very early in development and is prevalent until the age of six and drastically decreases later [32]. For the item “Betty is a soap bubble”, Child S said: “Betty has a soap bubble”, as if the “to be” condition could be reduced to the “to have” condition, which is actually equivalent to a literal interpretation. The only item Child S interpreted at a metaphorical level is “The prison guard is a rock”, where he explained that ”the guardian stands still”. This interpretation reflects a comparison based on a concrete attribute. At the immediately following item (“Family is an umbrella”), Child S provided an identical answer: “Family stands still like an umbrella”. This perseverative tendency also emerges in the APL Medea. For instance, in the Implicit Meaning Comprehension subtest, Child S either repeats the same answer from one item to the other or reproduces pieces of the question instead of providing answers to the question. Additionally, in the Situations subtest, Child S revealed his evident difficulty in inferring the different meanings a sentence may acquire when the context changes.

The poor performance of Child S on Colors Game, in contrast, with his reportedly fluent speech, deserves attention. His explanation of the rules of the game is limited to the following: “You cast the dice, a color must come out and you have to walk, and then, if you arrive first at the red (box), then you have won. If the yellow or the green come out you can’t move but if it is the black one, you stand still”. This type of answer shows that the child not only overlooks the majority of the criteria of this game (see above, par. 2.2), but more importantly, he disregards the main presuppositions concerning the listener to which he is explaining the game. For instance, Child S’ first sentence starts with the definite article “the”, associated to “dice”, taking for granted that the listener knows that dice are part of the materials of the game. This reaction might depend on Theory of mind capabilities, where Child S performs low.

Concerning Child D, his answers at Metaphor Comprehension Test are split into two halves: Six at zero level, i.e., literal, and six between level one and two, i.e., metaphorical. In his protocol, the type of zero level answers also stand out, especially considering his age. For instance, at the item “Friendship is a coat”, Child D says: “Friendship doesn’t last long”. Visibly, he limited himself to analyzing only the first of the two terms of the metaphor, completely eluding the comparison between the two.

On the APL Medea’s Metaphor subtest, all the performances were much under average, in both the subparts and the total score. For instance, the Iconic Metaphors subtest requires matching a given idiom (e.g. “To have one’s head in the clouds”) with an image that is supposed to represent it. In this case, the metaphorical interpretation is an image representing Donald Duck who is about to fall into a manhole. In this item, Child D chose the image that corresponds to a literal interpretation, i.e., Mickey Mouse with clouds pictured on his forehead. Some weaknesses also emerged in the Implicit Meaning Comprehension subtest. For instance, at the restaurant item (see Introduction), this is the way Child D answered the five questions posed in this item. (1) Concerning the physical place where the scene takes place, he said: “At home”; (2) What Mark is about to do, he answered: “He is about to eat”; (3) Who is talking, aside from Mark and Louise, he said: “It’s the butler”; (4) How will Mark come back home, he answered: “Walking”; and (5) What does Louise mean when she says “If you had to behave this way”: he answered: “Mark was wrong”.

These difficulties seem to be consistent with Child D’s Theory of mind capabilities. His performances on NEPSY II, which was much under what was expected at his age, highlights hesitations in items that require inferring someone’s thought concerning someone else’s thought.

Overall, when handling the meanings of the materials of the tests, both Child S and Child D showed significant misunderstanding. On the other hand, each profile revealed different types of strengths and weaknesses. For example, Child S performed poorly on the Metaphor Comprehension test and adequate on the APL Metaphor subtest, a discrepancy that can be explained by taking into account both the materials and the assessment modalities of these two tests. Child S’ good performance on the APL might reflect the fact that the majority of the items are idioms and that the Iconic Metaphor subpart does not require any verbal explanation but is simply to choose among pre-coded iconic representations of meanings. Child S might find it easier to retrieve conventionalized meanings, such as idioms, and recognize a metaphorical meaning in a multiple-choice task, rather than constructing new meanings by himself. Concerning Child D, in spite of an apparently satisfactory VCI (110), it is to be noted that nearly all his performances on the APL are under the expected level, except for the Conversational Strips subtest, which is consistent with his low performance at the Metaphor Comprehension Test.

## 5. Conclusions

The two profiles presented in our study underline the importance of systematically monitoring language development in individuals with KS, even when no delay is observed at early stages. Actually, for typically developing children, advances in language development bring to light more sophisticated capabilities that constantly require verbal inferencing beyond vocabulary knowledge and grammar rules. When these capabilities are called into play, individuals like Child S and Child D are challenged, which is likely to have heavy consequences on both academic proficiency and social adaptation in everyday interactions. This is particularly evident when we consider the key role of inferential processes in text comprehension [33,34] and conversation [35,36]. Given his low performance on the Implicit Meaning Comprehension and Situations subtests, Child S is very likely to experience difficulties in extracting meanings from implicit information and disambiguating polysemic words in a written text. In addition, based on his poor performance on the Theory of mind test, Child S might find it difficult to grasp the writer’s perspective. Child D, who performs even lower on the Theory of mind test and Implicit Meaning Comprehension subtest, would have as many troubles in constructing an overall text representation when reading. While both children seem to find no obstacles in turn-taking, based on their score on Conversational strips subtest, their poor performance on the Metaphor comprehension test and Colors game subtest suggests a precise difficulty in considering the other’s perspective in everyday conversation.

For this reason, overlooking the monitoring of these competencies across ages may represent a serious developmental risk.

Future research should delineate more in detail the developmental trajectories of pragmatic skills in children with KS, as pointed out by Ross and colleagues [3], and investigate the multiple factors that might account for their poor performance in this area. Insufficiently consolidated basic language abilities, deficits in attention, executive function, and Theory of mind may be responsible for these weaknesses. In addition, the interplay of these factors might also vary in each profile. Moreover, as pragmatic skills are based on inferential processes, as highlighted in the Introduction, i.e., on deductive operations from premises, themselves consisting in a multitude of contextual data, cultural aspects play a crucial role. Thus, monitoring the developmental trajectories of these abilities will also require explore the way cultural and educational stimulations can contribute to this development.

On clinical grounds, interventions can be envisaged to enhance pragmatic skills similarly to those already implemented with other clinical populations such as children with language disorders, learning disabilities, and autism spectrum disorder [37,38,39]. For example, treatments to enhance figurative language comprehension through explicit teaching of strategies are attested in literature [21,40,41]. The outcomes of these interventions might stimulate further experiences focused on children with KS to help them go beyond the literal meaning of words.

## Figures and Tables

**Table 1:** Raw scores, T scores (Metaphor Comprehension Test: MCT) and scalar scores (NEPSY II).

	MCT T Score	NEPSY II Theory of Mind Scalar Score	NEPSY II Affect Recognition Scalar Score
Child S	(1) 25	(18) 7	(26) 10
Child D	(10) 32	(20) 5	8

Legend: Scores in bracket are raw scores and scores without bracket are T or scalar points. Max total score: MCT = 36; ToM = 25; and Affect Recognition = 35.

**Table 2:** APL Medea subtests and total test. Raw scores and z scores.

	APL Medea							
	Verbal Metaphors	Iconic Metaphors	Metaphor	Implicit Meaning Comprehension	Conversational Strips	Situations	Colors Game	Total APL
Child S	(3) −0.71	(4) 0.53	(7) −0.05	(6) −1.17	(6) −0.47	(1) −2.47	(3) −2.04	(23) −1.59
Child D	(2) −2.58	(2) −1.80	(4) −2.80	(8) −1.75	(12) 1.22	(6) −1.00	(7) −1.39	(37) −2.18

Legend: Scores in bracket are raw scores and scores without bracket are z scores. Max total score: Metaphor = 16; Implicit Meaning Comprehension = 14; Conversational strips = 12; Situations = 8; Colors game = 15; and Total APL = 65.
